# The results of nasopharyngeal cancer patients treated by simultaneous integrated boost technique and concomitant chemotherapy

**DOI:** 10.3906/sag-1605-98

**Published:** 2019-04-18

**Authors:** Mehmet Faik CETİNDAĞ, Atiye Yılmaz ÖZSAVRAN, Bülent YALÇIN, İclal ÇETİNDAĞ, Karabekir ERCAN, Şeyda TÜRKÖLMEZ, Dinçer YEĞEN

**Affiliations:** 1 Radiation Oncology Clinic, Atatürk Training and Research Hospital, Ankara Turkey; 2 Radiation Oncology Clinic, Elazığ Training and Research Hospital, Elazığ Turkey; 3 Medical Oncology Clinic, Atatürk Training and Research Hospital and Yıldırım Beyazıt University Hospital, Ankara Turkey; 4 Radiology Clinic, Zekai Tahir Burak Training and Research Hospital, Ankara Turkey; 5 Radiology Clinic, Atatürk Training and Research Hospital, Ankara Turkey; 6 Nuclear Medicine Clinic, Ankara Atatürk Training and Research Hospital and Yıldırım Beyazıt University Hospital, Ankara Turkey; 7 Department of Medical Physics, Ankara Atatürk Training and Research Hospital, Ankara Turkey

**Keywords:** Nasopharyngeal carcinoma, helical tomotherapy, concomitant weekly chemotherapy

## Abstract

**Background/aim:**

To assess the efficacy and side effects of concurrent weekly chemotherapy and radiotherapy with simultaneous integrated boost (SIB) technique for nasopharyngeal cancer (NPC).

**Materials and methods:**

A total of 51 consecutive patients with diagnosis of NPC were treated between February 2010 and December 2015. The median dose for PTV70 (range: 60–82) was given in 33 fractions (range: 31–35). Forty-five of the patients received concomitant weekly chemotherapy between 3–7 cycles (median 6). Eleven patients received neoadjuvant and thirty-nine patients received adjuvant chemotherapy.

**Results:**

At a median follow-up 43 months (range: 2–76) the estimated 5-year overall survival and disease-free survival were 74.6% and 62.6%, respectively.

**Conclusion:**

In radiotherapy of advanced NPC, generally a considerable amount of normal head and neck tissues might have to be irradiated in addition to gross tumor volume, involved node, and elective neck irradiation. Together with chemoradiotherapy, poor oral hygiene and inadequate nutritional support result in excessive morbidity. Despite loco-regional success of concurrent chemoradiotherapy, distant metastasis is still the major pattern of treatment failure in the intensity modulated radiotherapy era. We need to improve our adjuvant chemotherapy regimens or develop new drugs.

## 1. Introduction

Nasopharyngeal cancer (NPC) arising from surface epithelial cells of the nasopharynx was first reported as different type of cancer by Regaud and Schmincke in 1921 (1). Compared with Asians, NPC is uncommon in western populations; the lowest ratio with an incidence of 0.2 to 0.5 cases per 100,000 people in the United States and the highest ratio with 20 to 50 cases per 100.000 people in southern China (2,3). In the Turkish population, the incidence is between these two extremes and most of the patients are middle-aged males with a low to median socioeconomic status. Fried meat is especially an important risk factor for the southeastern Anatolian region of Turkey (4). The big variations among geographic regions and ethnic predisposition indicate the complex nature of etiology and the interaction of genetic, viral, environmental, and dietary risk factors (2,3). Preliminary studies on plasma Epstein-Barr virus (EBV) DNA proved that it can be a promising screening tool for early detection of NPC in endemic areas of southern China. In addition, EBV DNA can be a powerful prognosticator of recurrence and survival as well as a strong complement for tumor burden and staging (5,6).

Three subtypes of NPC are recognized in the World Health Organization (WHO) classification: Type 1, squamous cell carcinoma, typically found in the older adult population; Type 2, nonkeratinizing carcinoma; Type 3, undifferentiated carcinoma. Most cases in childhood and adolescence are Type 3, with a few Type 2 cases. Type 2 and 3 are associated with elevated Epstein-Barr virus titers but Type 1 is not (2,3,5,6).

Upper cervical lymph adenopathy is the initial presentation in many patients. Symptoms related to the primary tumor include epistaxis, nasal obstruction, and discharge. Cranial nerve palsies due to skull base involvement lead to a variety of symptoms. Distant metastasis, at which the bone is most affected, is seen in 3% to 6% of the cases at presentation and may occur in 18% to 50% of the cases during the disease course (2,3).

Because of the anatomic location and vicinity to critical structures, tumor resection without excessive morbidity is very challenging in NPC (7,8). Primary surgical intervention of NPC was abandoned in 1950. Treatment strategy should be tailored to 3-dimensional conformal radiation therapy (3-DCRT) and recently intensity modulated radiotherapy (IMRT). While single-modality therapy is adequate for the early stages of NPC (I, II), combined-modality therapy is necessary for advanced stages (III, IV) (2,3). It has not yet been proved whether induction or adjuvant chemotherapy adds further benefit to CCRT. It remains to be investigated (2,3,9–15).

The development of IMRT has greatly eliminated the shortcomings of traditional radiotherapy techniques (16–21). With IMRT, not only the dose coverage and conformity to the tumor target area improved but also the damage to normal tissues, particularly to the salivary glands, is reduced. IMRT significantly ameliorates xerostomia and prevents a decrease in salivary secretions. Nevertheless, more than 30% of IMRT-treated NPC patients still suffer from long-term side effects. A newly developed technique, helical tomotherapy (HT), combines inverse planning of intensity modulation and a full 360-degree radiation beam direction, resulting in better dosage conformity, higher tumor control probability (TCP), and lower normal tissue complication probability (NTCP). 

## 2. Materials and methods

### 2.1. Patient characteristics

Between February 2010 and December 2015, 51 consecutive patients with newly diagnosed NFC were treated with image guided (IG)-IMRT. All patients including one with distant liver and bone metastasis at presentation were evaluated in this study. Patient characteristics are indicated in Table 1. The pretreatment evaluation consisted of flexible fiberoptic nasopharyngoscopy examination, blood chemistry tests, computed tomography (CT) scans, magnetic resonance imaging (MRI), and dental evaluation. 18-Fluoro-deoxyglucose positron emission tomography (FDG-PET) for the purpose of radiotherapy planning and distant metastasis evaluation was obtained in 38 of the 51 patients. The median age was 50 years (range: 9–78 years). Of the total 51, 41 patients were male. The distribution of clinical stages, according to the American Joint of Cancer Committee, was 3 patients (5.9%) at stage I, 12 (23.5%) at stage II, 13 (25.5%) at stage III, 11 (21.6%) at stage IVA, 11 (21.6%) at stage IVB, and 1 (2%) at stage IVC. T1, T2, T3, and T4 diseases were found in 15 (29.4%), 16 (31.4%), 6 (11.8%), and 14 (27.5%) patients, respectively. N0, N1, N2, and N3 diseases were found in 9 (17.6%), 14 (27.5%), 17 (33.3%), and 11 (21.6%) patients, respectively. According to the ECOG performance scale 13, 29, and 9 patients have 0, 1, and 2 performance status, respectively. The most frequently seen symptoms at presentation were a neck mass in 24 (47%) patients, hearing loss or ear drainage in 14 (27%) patients, nasal bleeding or obstruction in 11 (22%) patients, headache in 6 (12%) patients, and swallowing problems or cranial nerve palsy in 6 (12%) patients. According to the WHO classification, pathologic variations of patients consist of 5 (9.8%) patients with Type I, 27(52.9%) with Type II, and 19 (37.3%) with Type III.

**Table 1:** Patient characteristics.

Characteristics	Number Range: median
SexFemale: Male	10: 41
Age	9- 78: 50
T-stageT1: T2: T3: T4	15: 16: 6: 14
N-stage N0: N: N2: N3Stage1: 2: 3: 4A: 4: 4C	9: 14: 17: 113: 12: 13: 11: 11: 1
Metachronous second cancersLung: Larynx: BladderMetastasis at diagnosisLiver Metastasis after treatmentBone: Liver: Mediastinal	1: 1: 114: 4: 1
PathologyWHO Type I: II: IIIECOG performance status0: 1: 2	5: 27: 1913: 29: 9
Neoadjuvant CTYes: NoConcomitant CTYes: NoAdjuvant CTYes: No	11: 4045: 637: 14
Symptom at presentationNeck massHearing deficits or ear dischargeNasal obstruction or bleedingHeadacheSwallowing problems and cranial nerve palsyWeight loss duringchemoradiotherapy<6: ≥6NeutropeniaGrade 0: 1: 2: 3Oral mucositisGrade 0: 1: 2: 3Temporal lobe necrosisHearing lossLeft side: right sideDeathNonrelated to NFCTreatment toxicityMetastatic and progressive diseases	2414116620: 3126: 1: 11: 11: 11: 26: 1213: 2236

### 2.2. Treatment: Chemotherapy

In our cohort, 11 patients received a median of 4 cycles of neoadjuvant chemotherapy (range 1–5), 45 patients received concomitant weekly chemotherapy with a median of 6 cycles (range: 3–7), and 37 patients received adjuvant chemotherapy with a median of 3 cycles (range: 1–6). Neoadjuvant chemotherapy consists of intravenous infusion of cisplatin at 75 mg/m2 on day 1 and continuous intravenous infusion of 5-fluorouracil (5-FU) at 800 mg/m2 on days 1 to 5 (120 h infusion) at 28-day intervals in 5 patients or the same protocol is followed with the addition of docetaxel at 75 mg/m2 on day 1 in 6 patients. CCRT was given by intravenous infusion of weekly cisplatin at 40 mg/m2 in 37 patients, or intravenous infusion of weekly carboplatin at 30 mg/m2 plus intravenous infusion of weekly docetaxel at 30 mg/m2 in 7 patients and weekly 75 mg/m2 carboplatin in 1 patient. Drug combination of adjuvant chemotherapy consists of cisplatin at 75 mg/m2 on days 1 and 8 and 5-FU at 600 mg/m2 on days 1 to 5, at 3-week intervals in 33 patients, carboplatin and docetaxel in 1 patient, and carboplatin and 5-FU in 3 patients. Cisplatin was decreased to 50 mg/m2 if the absolute neutrophil count was 1000–1500 cells per μL, platelet count was 50,000–75,000 per μL, or creatinine clearance was 40–60 mL/min. Chemotherapy was stopped if the creatinine clearance was less than 40 mL/min.

### 2.3. Treatment: Radiotherapy

All patients were immobilized in the supine position using a custom-made thermoplastic cast from head to shoulders (Klarity Medical Equipment Co.). The 3-D volumetric scans (true point PET-CT, Biograph, Siemens) with contrast and without contrast were acquired at 3-mm slice thickness. The CT images were transferred to a virtual simulation workstation (TomoCon 3.0 TetraMed) for registration and structure delineation. For every patient, MRI with gadolinium contrast was obtained and registered to the planning images. Of the 51 patients, 36 had also PET-CT images, which were obtained in the same fixed position with thermoplastic masks and also registered to the planning CT image dataset for contour delineation. The target volumes were defined by the guidance of the ICRU 50 and 62. The primary gross tumor volume of nasopharynx (GTVnp) and involved lymph nodes (GTVln) were delineated out on each slice with the fusion of MRI and PET-CT images. For patients who received neoadjuvant chemotherapy, the prechemotherapy volume of the nasopharyngeal extension was used for GTVnp delineation and postchemotherapy volume of lymphatic involvement was based on GTVln delineation. Four clinical target volumes (CTV) were defined in our clinic; CTV1was defined as the GTVnp plus 3–10 mm margin and CTV2 was defined as the GTVln plus 3–7 mm margin to encompass high risk sites of microscopic extension. CTV3 was defined to cover the high risk subclinical region at the primary disease site, which includes the entire nasopharynx, anterior 1/2 to 2/3 clivus, skull base (foremen ovale and rotundum bilaterally), parapharyngeal space, inferior sphenoid sinus, pterygopalatine fossae, posterior fourth to third of the nasal cavity, maxillary sinuses, and the high risk subclinical region at the lymph nodal regions, which include bilateral retropharyngeal, bilateral level II, level III, and level VI lymph nodes. CTV4 was defined as bilateral level IV lymph nodes. At the discretion of the radiation oncologist, level IB might also be spared or limited to the anterior border of the submandibular gland in low risk node positive patients. We also note that the outer most boundary of CTV2 should be at least 15 mm from the GTVnp. The exception of that rule was complete tumoral infiltration of clivus and tumoral formation adjacent to the brain stem, chiasma, or optic nerves. In those situations, the margin could be as small as 1 mm. To obtain PTVs, we enlarged CTVs 1 to 3 mm in all directions. The spinal cord, brain stem, optic chiasm, optic nerves, eyeballs, the lenses, temporal lobes, parotid glands, oral mucosa, larynx, and temporomandibular joints were delineated as the organs at risk (OAR). Treatment plans were computed using a commercial serial tomotherapy planning system (TomoTherapy Planning Station Hi-Art Version 4.2.2). IMRT was delivered with 2.5 cm field width, pitch of 0.287, and modulation factor of 2.5. Mean PTV for tumor was 131 cm³ (range: 27–351). Mean PTV for involved lymph nodes was 116 cm³ (ranges 16-598). Mean PTV 60 Gy was 511 cm³ (range: 72-887). Mean PTV54 Gy was 149 cm³ (range: 20–345). All plans should be normalized such that at least 95% of the volume of the PTV70 is covered by the 70 Gy isodose surface. The prescribed dose was 70 Gy to the PTV1, 66 Gy to the PTV2, 60 Gy to the PTV3, and 54 Gy to the PTV4 in 33 fractions. The calculated mean PTV doses 70 Gy, 66 Gy, 60 GY, and 54 Gy were 70.3 Gy, 66.4 Gy, 63.6 Gy, and 53.9 Gy, respectively. Simultaneous integrated boost (SIB) technique was applied by helical tomotherapy (HT) with one fraction daily over 5 days per week. Volumetric image guidance by mega voltage computed tomography (MVCT) scanning was routinely applied for every fraction of all patients. Adaptive radiotherapy was planned in 18 patients because of lymph node shrinkage and weight loss. The total radiochemotherapy delivery time ranged from 42 days to 65 days (median 48 days). Radiotherapy characteristics are given in Table 2.

**Table 2:** Radiotherapy characteristics.

Characteristics	Numberrange: median
Prescribed total dose (Gy)Fraction dose for PTV 70: 60: 66: 54Mean PTV 70: Volume cm3Mean PTV 66: Volume cm3Mean PTV 60: Volume cm3 Mean PTV 54: Volume cm3Number of fractions	60–82: 702.17: 1.82: 2: 1.6327–351: 13116–598: 11672–887: 51120–345:14931–35: 33
Duration of concomitant chemoradiotherapy (days)	42–65: 48
Brachytherapy boostAdaptive planningYes: No	120: 31

All patients were evaluated weekly for treatment response and toxicity during chemoradiotherapy. After completion of radiotherapy, the first follow-up was done within a month by using indirect fiberoptic nasopharyngoscope and physical examination, thereafter at 3-month intervals for the first year, at 4-month intervals for the second year, and at 6-month intervals after that. Each follow-up included a detailed questioning of early and late side effects, clinical examination of head and neck, complete blood, biochemical, renal and liver profile, indirect fiber optic nasopharyngoscope examination, MR of nasopharynx and neck, CT or ultrasound of abdomen, chest X-ray, and, where necessary, PET-CT, bone scan, and biopsy. Toxicity assessment was made according to the National Cancer Institute’s Common Terminology Criteria for Adverse Events (NCI CTCAE 3.0). 

The follow-up period was measured from the first day of adjuvant chemotherapy or chemoradiotherapy. SPSS 18 (SPSS Inc., Chicago, IL, USA) was used for statistical analysis. The endpoints for early toxicity were death from the neutropenia, infections, and malnutrition. The end points for tumor control include actuarial rates of local-nodal failure-free rate (LN-FFR: persistence/recurrence in the nasopharyngeal or lymphatic region), distant metastasis-free survival (DMFS: duration of survival without distant metastases), and disease-free survival (DFS: survival without distant and local failure). Disease-specific survival (DSS: censoring deaths not due to NPC) and overall survival (OS: duration from the beginning date of therapy to the date of death) were measured from any cause or the censoring of the patient at the date of last follow-up. The actuarial rates were calculated by Kaplan–Meier method and the differences were compared by the log-rank test. A separate log-rank test was used to identify the independent risk factors on survival. A 5% Type-1 error level was used to infer statistical significance.

## 3. Results

We assessed 51 consecutive NFC patients. No patient was excluded from the study for any reason. Two loco-regional recurrences were observed during the median (43 months) follow-up time. One patient had liver metastases at presentation and three patients already had metachronous second cancers (bladder, larynx, and lung). The patient with liver metastases at diagnosis responded to curative intent therapies. He lived more than 25 months after metastases and there was no evidence of the loco-regional relapse when he died. One patient with T1N0M0 had suspicious nasal mass appearance on MR 3 months after completion of radiotherapy and brachytherapy boost. He was given 70 Gy in 2.17 Gy fractions by tomotherapy, followed with 3 Gy brachytherapy boost four times in another institute. F-18 FDG PET-CT conducted 4.5 months after RT showed a 1 × 2 cm mass lesion, which had an increase of uptake in the late phase (SUV(max): 9), but 2 biopsies obtained a 2-month interval by using indirect nasopharyngoscope demonstrated granulation tissues and the patient was in good condition with no recurrences and no late complication five years after treatment. One patient with distant metastasis (liver) has been given multiagent chemotherapy. 

**Figure 1:**
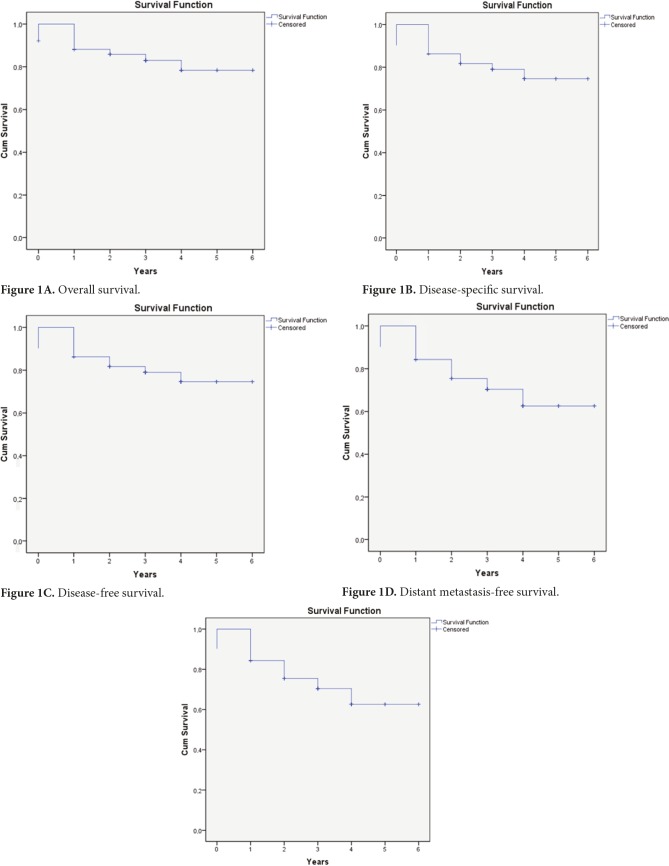
Local-nodal failure-free survival.

The 5-year rate of OS, DSS, DFS, DMFS, and LN-FFR are calculated as 74.6%, 78.4%, 62.6%, 74.6%, and 65.9% and are plotted in Figures 1A, 1B, 1C, 1D, and 1E, respectively.

**Figure 2:**
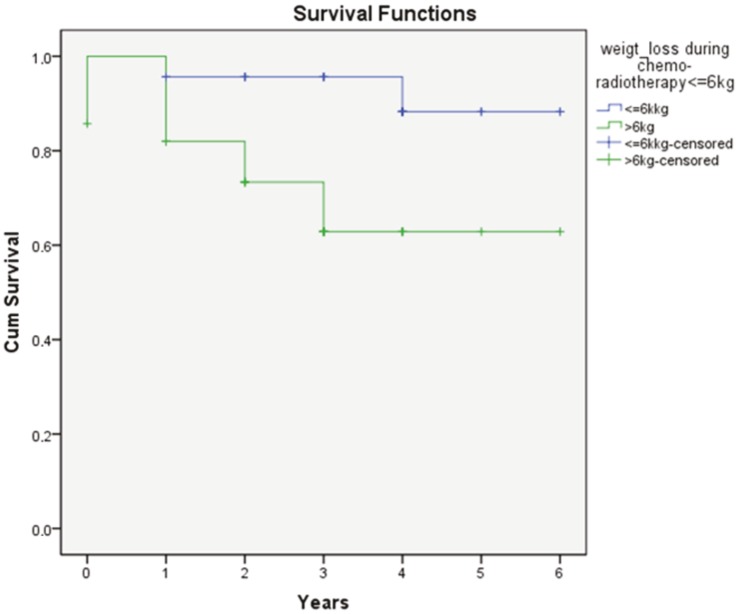
Weight loss of less than 6 kg has a significant survival advantage on DFS. Log-rank (Mantel–Cox) (P = 0.026).

Sex, age, ECOG performance status, T-stage, N-stage, stage, the presence of neutropenia, type of concomitant chemotherapy (CCT) used, and weight loss during CRT were examined using the log rank test (Mantel–Cox). Among the investigated risk factors, weight loss of more than 6 kg during CRT was found statistically significant for DFS (P = 0.029; Figure 2). The most common acute toxicity was oral mucositis, which caused treatment interruptions in 13 patients in 2–15 days (median 5 days). One patient died because of severe neutropenia during CCRT. From the retrospective medical record examination of this patient, we found that he had lost 30 kg (from 96 to 66 kg) in 50 days. He had refused a nasogastric tube feeding or percutaneous endoscopic gastrostomy tube placement during CCRT. His concomitant chemotherapy regime consisted of weekly cisplatin at 30 mg/m2 and weekly docetaxel at 30 mg/m2. One patient died after the 25th day of CCRT because of malnutrition and systemic infections. One patient died because of toxicity during the first session of adjuvant chemotherapy. Six patients under systemic chemotherapy with distant metastases died due to progressive disease at 5, 14, 29, 31 46, and 56 months after completion of CCRT. Two patients’ death was nonrelated to cancer progression; one was 14 months later due to heart attack, and the other was 29 months later due to intracranial hemorrhages. A unilateral temporal lobe necrosis was developed in one patient, after 36 months from the CCRT. She had been operated on and histologic verification of radiation necrosis was demonstrated. She was given anticonvulsants and was in good condition after the operation. Grade I and II xerostomias were observed in 26 and 17 patients, respectively. Four patients had moderate unilateral hearing loss and one patient had elevated thyroid stimulating hormone.

## 4. Discussion

After demonstration of survival benefits for CCRT versus 2-D and 3-D conformal RT alone in a pivotal study by Muhyi Al-Sarraf et al. (14), which was carried out in 1998, attention among NPC investigators turned to CCRT in the next decade. In this phase III randomized study, they showed that 3-year survival rate was 47% in the RT arm compared to 78% in the CCRT arm (P = 0.001). In this era, we also witnessed tremendous improvements in radiotherapy techniques and extraordinary developments in radiologic and nuclear medicine imaging. While F-18 FDG PET/CT is giving us information about local and distant metastases and thus correct staging, MR led us to define correct demarcation of local tumoral invasion. In collaboration with sophisticated IG-IMRT and modern imaging techniques we had substantially increased loco-regional control rates in this complex anatomical region. The 5-year survival rates for NPC increased gradually from 50% in 1960–1970s to 80% in 2000–2010s, and an average 30% improvement obtained in this half-century period. Local and regional failures are no longer a big issue for NPC, but distant metastases are still the most commonly seen failure pattern after treatment with IMRT and concurrent chemotherapy. 

A total of 11 patients died in our cohort. Three deaths occurred in the vicinity of concurrent chemoradiotherapy period. These were accepted as treatment related toxicity mortalities. It is unacceptable in terms of morbidity and mortality. Except one, all the other mortalities occurred in the post-CCRT period. One patient who died with febrile neutropenia in the CCRT period had lost 30 kg of his pretreatment weigh (from 96 to 66). He refused to get supplementary food in the beginning of therapy and also did not want nutritional support by nasogastric tube feeding or percutaneous endoscopic gastrectomy tube feeding. The febrile neutropenia remains a potentially life-threatening complication that requires prompt medical interventions. In our case, immediate hospitalization and rapid initiation of broad-spectrum antibiotics therapy were not given because of insufficient emergency care units. The other two mortalities that occurred within 2 months after CCRT were accepted as toxicity related deaths. One patient had severe oropharyngeal mucositis with fungal infections and died because of sepsis; the other had long duration filgrastim resistant neutropenia during the first session of adjuvant chemotherapy. We thought that aggressive CCRT and insufficient supportive care resulted in these unacceptable mortality rates. None of the patients who died because of treatment related toxicity had persistence, recurrence, or loco-regional progressive disease. We had four liver, one lung, and two bone metastases during the follow-up period. Four metastatic patients died because of progressive diseases under chemotherapy. One of them had also loco-regional recurrences. In our study, T and N stage did not have significant prognostic importance for recurrence and loco-regional failure, probably because of adequate coverage of irregularly shaped tumors by IMRT. IGRT (megavoltage computed scanning for every fraction) also adds an important contribution to the precision and reliability of radiotherapy. In addition, aggressive concomitant chemotherapy had an important part in this success. However, in our cohort, 5 out of 51 nonmetastatic NPC patients still developed distant metastases. PET-CT images of this metastatic NPC revealed no loco-regional recurrences. While CCRT has a significant impact on loco-regional treatment of NPC, the role of adjuvant systemic chemotherapy needs to be investigated. To fight long-term distant metastasis of NPC, we have to improve our adjuvant chemotherapy regimens or develop new drugs.

In a Phase III study of adjuvant chemotherapy in advanced NPC patients, Chi et al. (11) demonstrated that adjuvant chemotherapy had no benefit for overall survival or relapse-free survival. In another IMRT study by Lee et al. (18), it was demonstrated that 1 local and 1 nodal failure but 17 systemic failures had occurred in a cohort of 67 patients. Baujat et al. (13) concluded that the overall and event-free survival contribution of chemotherapy came from the concomitant use, not the adjuvant one, in their analysis of eight randomized trials, which consisted of 1753 NPC patients. In their matched cohort analysis, Chua et al. (12) demonstrated that CCRT improved loco-regional control in Chinese patients with loco-regionally advanced NPC, but their analysis failed to detect any impact on distant failure and survival. Sun et al. (19) published long-term results of their IMRT series with 868 patients. They argued the role of CCRT in patients with stage III/IVA-B disease and found that CCRT added no survival or local control advantage in this group of patients. Lee et al. (24) in their study assessed the therapeutic gains and setbacks from the 2-dimensional radiotherapy to conformal 3DRT to IMRT era. They concluded that significant improvements in survival and reductions of serious toxicity were obtained during this period and the average 5-year survival rates for NPC increased steadily from around 30% in 1960–1970, 50% in 1980, and 70% in the 1990s. This study further showed 85% DSS in the IMRT era, with reductions of late neurological toxicity. We had one temporal lobe necrosis, four cases of hearing loss, and one TSH elevation and we believe that these rates were in the acceptable limits. Despite acute grade V toxicities, our 5-year estimated overall survival (74.6%) and disease-specific survival (78.4%) rates are within the proportions of the IMRT era. Because we have only two loco-regional recurrences and our major loss is due to acute treatment toxicities and progressive distant metastatic disease, we conclude that loco-regional recurrences are no longer a big issue of NFC treatment. We also did not find any statistically significant difference among N-stage and T-stage in Kaplan–Meier survival analyses. This is probably due to insufficient number of patients in every group of stage in our cohort. The compliance rate to chemotherapy was good in our series as three, one, eight, twenty-four, and eleven out of 45 CCRT patients completed 3, 4, 5, 6, and 7 weeks of concomitant chemotherapy cycles, respectively. A high level of compliance to chemotherapy and good coverage of GTVs with high dose seem important benchmarks of loco-regional success in NPC. IMRT is also capable of reducing doses to the salivary glands, temporal lobes, cochlea, optic apparatus, and brain stem and thus preventing sequelae from early radiotherapy. Permanent xerostomia results in compromised quality of life due to eating, speaking, and swallowing problems. It also causes dental caries, infections, ulcers, and, in worst cases, osteoradionecrosis. In our series 20 patients have adaptive planning because of weight loss and lymph node and parotid gland shrinkage. Adaptive planning provides an important contribution in decreasing of parotid doses. As parotid gland dose decreases, its improvement increases gradually in time. In our series, most grade I–II xerostomia patients had near-complete recovery in their salivary function at the end of one and half years. Despite loco-regional success of IMRT, distant metastasis is the major pattern of treatment failure in NPC.

In conclusion, more effective and less toxic treatment modalities need to be investigated in the near future to reduce distant metastatic failure in NPC.
